# Re-contact demographics and clinical characteristics of diabetic patients treated for a hypoglycaemic episode in the pre-hospital environment: a rapid literature review

**DOI:** 10.29045/14784726.2019.09.4.2.10

**Published:** 2019-09-01

**Authors:** Karl Bloomer

**Affiliations:** Northern Ireland Ambulance Service HSC Trust: ORCID iD: https://orcid.org/0000-0002-7822-4528

**Keywords:** diabetes, hypoglycaemia, re-contact

## Abstract

**Introduction:**

Diabetes mellitus has been referred to as an ‘epidemic’ and the World Health Organization reported 422 million people with the disease in 2014. Hypoglycaemia is common among emergency presentations, yet understanding around the utilisation of emergency medical services (EMS) for this is incomplete.

Ambulance service referral pathways for those suitable to be treated in the community have been developed as a means of managing this growing demand. However, there is limited evidence to suggest how they should be constructed or implemented.

The aim of this review was to examine patients who re-contacted the health services following EMS non-transport for a hypoglycaemic episode and to determine if risk factors could be identified.

**Methods:**

Medline/PubMed and CINAHL online databases were searched for papers published between 1998 and 2018 relating to re-contacts following an interaction with EMS. The Cochrane Library online database was also searched, as well as manual searches from key journals. Relevant clinical manuals, guidelines and specific grey literature were also hand searched.

**Results:**

After duplicates were removed, 260 articles were identified, with 41 selected for full review. These were then reduced by excluding those that did not provide any data on re-contact rates/demographics. The remaining papers were then assessed using the Critical Appraisal Skills Programme (CASP) appraisal tool and those identified as of low quality were removed. This produced 17 papers for final inclusion.

**Conclusion:**

The literature demonstrates that ambulance clinicians can appropriately treat hypoglycaemia in the community and identify those requiring further assessment at emergency departments. However, due to the very nature of diabetes, repeat episodes will and do occur, regardless of community or emergency department management, but these are rarely in the acute phase. Some groups are higher risk, but thorough holistic assessment is vital for identifying those suitable for community management.

## Introduction

Diabetes mellitus has so extensively spread around the world’s populations that Cypress and Gleeson (2009) refer to it as an ‘epidemic’ and the World Health Organization (2017) reported 422 million people with the disease in 2014.

Within this group, hypoglycaemia is common among emergency presentations, and patients diagnosed with type 1 diabetes commonly experience one to two mild episodes a week, with around at least one severe episode a year (Brackenridge, Wallbank, & Lawrenson, 2006; Cryer & Arbelaez, 2017; Hirsch, 2009). However, understanding around the utilisation of emergency medical services (EMS) in the pre-hospital setting for hypoglycaemia is incomplete and, as Villani et al. (2016) point out, reported figures can vary widely from 0.6 to 4.7% of total attendances.

As a means of more appropriately managing this growing patient group, ambulance service referral pathways were developed as part of a UK wide project aimed at reducing the number of avoidable hospital journeys (Snooks et al., 2004). However, there remains little evidence or guidance to suggest how they should be constructed or implemented (Bell & Fitzpatrick, 2016; Department of Health, 2005).

The aim of this review was to examine data surrounding those patients who re-contacted a form of healthcare following EMS non-transport/discharge/community referral for a hypoglycaemic episode and to determine if risk factors could be identified.

## Methods

A scoping literature review was performed to explore pre-hospital hypoglycaemic management and re-contact rates. Medline/PubMed and CINAHL online databases were searched using the following criteria: (paramedic OR ambulance OR prehospital OR pre-hospital OR EMS) AND (Hypoglyc* OR low blood sugar OR low blood glucose). The Cochrane Library online database was also searched using the same criteria as above and variations of the same. Additionally, manual searches were undertaken from key journals including *Emergency Medicine Journal*, *British Paramedic Journal* and *Journal of Paramedic Practice*. The majority of studies were located somewhere around the midway point of the evidence hierarchy and to exclude them would have resulted in a very limited number of papers for review; therefore all levels of evidence/study design were included for screening and appraisal.

Articles published between 1998 and 2018 were screened initially by title and then abstract. Papers were excluded if they were not in English or from a peer-reviewed publication. Those not freely available or accessible through the researcher’s existing database privileges were also excluded. After a full review these were then reduced by excluding those that did not provide any data on re-contact rates/demographics. The remaining papers were then assessed against the methodologically relevant Critical Appraisal Skills Programme (CASP UK, 2019) appraisal tool. The total score of positive results was calculated and any articles that did not score at least 50% in their relevant CASP checklist were subsequently rejected on the basis of quality.

Relevant clinical manuals and guidelines including the IHCD *Ambulance service paramedic training manual* (Ambulance Service Association, 1999), the *UK ambulance services clinical practice guidelines 2016* (Joint Royal Colleges Ambulance Liaison Committee & Association of Ambulance Executives, 2016) and grey literature in the form of several UK ambulance service diabetic hypoglycaemic treat, leave and refer procedures were also hand searched.

## Results

**Figure 1:**
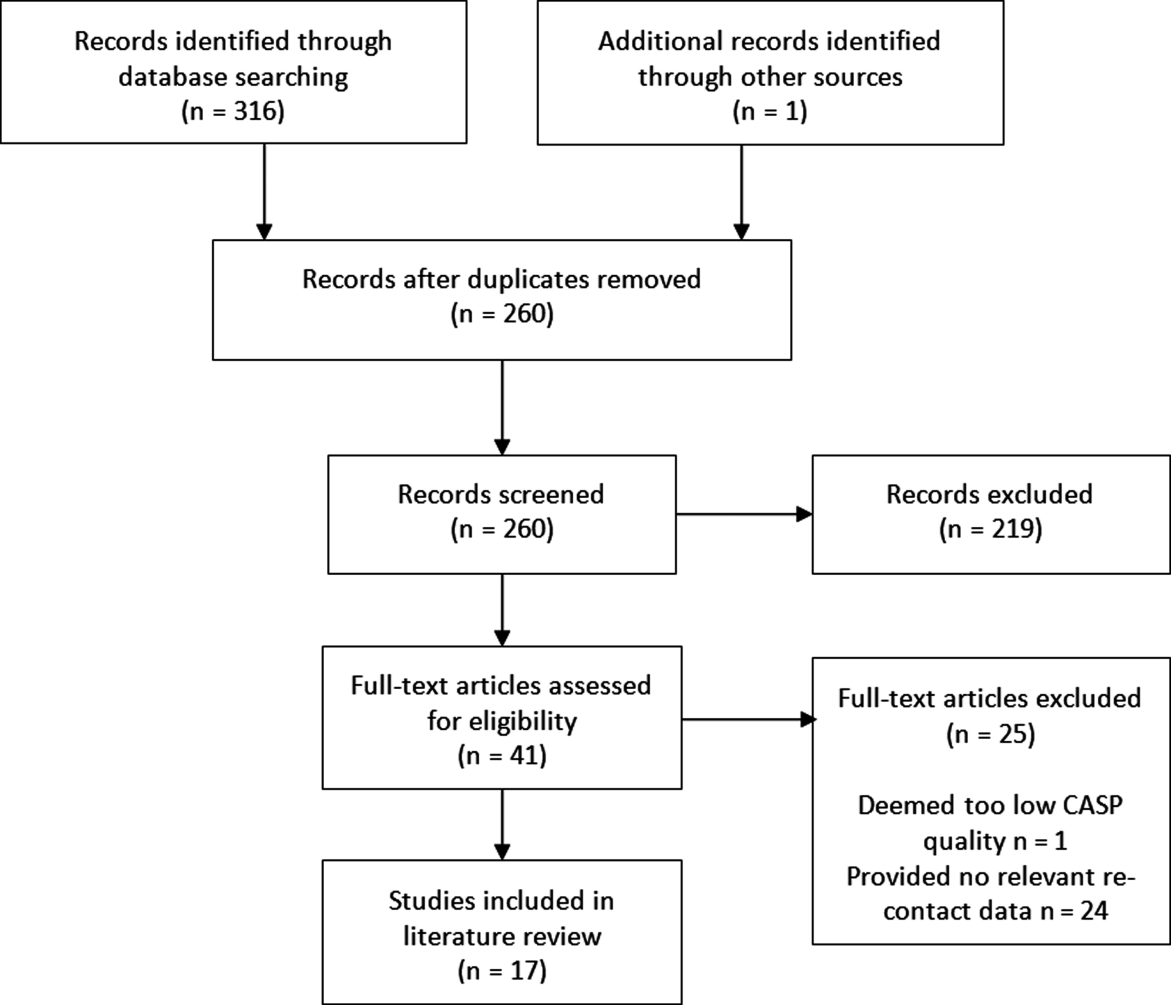
Figure 1. PRISMA diagram of searched literature.

Note: CASP = Critical Appraisal Skills Programme.

After duplicates were removed, 260 articles were identified, with 41 selected for full review (Figure 1). After a full review, these were then reduced by excluding those that did not provide any data on re-contact rates/demographics or were of insufficient quality. This produced 17 papers for final inclusion (Table 1).

**Table 1.** Summary of included studies.

Article	Study type and length	Number included in study	Re-contact rates	Non-transport figures (patients left at scene)	Summary of key findings
Sinclair et al. (2018)	Retrospective cohort study *(Jan 2011–Dec 2011)*	791	Non-transported patients < 72 hrs EMS: 2.1% (n = 5) All H/C: 3.8% (n = 9) All patients < 72 hrs EMS: 3.5% (n = 28) All H/C: 5.4% (n = 43)	29.7% (n = 235)	Refusal of transport decisions only in EMS system. Those not transported to ED were younger and more likely to be on insulin. Although data on type of diabetes incomplete, a trend showed T1 more likely to not be transported. Patients with certain comorbidities more likely to re-contact. Sulphonylureas were not a predictor of re-contact.
Sampson et al. (2017)	Prospective cohort study *(Dec 2014–Apr 2016)*	2000	18.6% (n = 372) reported previous 999 ambulance attendance in previous month for a SHE	86.2% (n = 1724)	T2 patients on insulin experience a mean of 1.05 SHE per year. Insulin treated DM more clinically severe episodes than oral hypoglycaemic treated group. 43.8% > 70 yrs old. Insulin error, missed meals and illness highest causes.
Bell & Fitzpatrick (2016)	Short-cut literature review *(1996–2006)*	N/A	2–7% < 48 hrs	38.2% (n = 107)	No significant difference in re-contact rates between transported and not transported groups. Authors conclude hypoglycaemic referral pathways may be of patient benefit, but insufficient evidence at present time to determine impact.
Tohira et al. (2015)	Retrospective cohort study *(Jan 2013–Dec 2013)*	609	0.5%–2.7% depending on 24 hrs/3 days/7 days and checklist criteria met or not	29% (n = 174)	Checklists identified significantly fewer patients for discharge at scene than paramedics actually discharged. No difference in subsequent events between the groups that met the checklists and those that did not. Checklists do not accurately identify those suitable for paramedic discharge; clinical judgement required.
Hatting & Mikkelsen (2015)	Retrospective descriptive study *(May 2006–Apr 2010)*	108	34% (n = 17) < 30 days 6% (n = 4) < 24 hrs	50% (n = 69)	Physician based model. Discuss possibility that T1DM may be only safe category of patient that could be discharged pre-hospital. Discussed risk of RHE in patients on oral antihyperglycaemic medications.
Khunti et al. (2013)	Retrospective case report *(Nov 2010–Feb 2011)*	523	0.5% (n = 2)	70% (n = 367)	Insulin treatment associated with highest frequency of EMS DM related calls. Insulin treated DM less likely to be transported to ED. Higher respiratory rate a statistically significant predictor for hospital transportation. No other outcome measures predicted transportation to hospital. Estimate SHE at rate of 2.76 per 100 person years. 28% nocturnally. Recurrent episodes and impaired awareness are major risk factors for SHE.
James et al. (2013)	Prospective cohort study *(Sep 2010–Mar 2011)*	47	17% (n = 8) admitted within 30 days 4% (n = 2) for hypoglycaemia related	Not reported	One re-contact direct result of sulphonylurea medication.
Parsaik et al. (2013)	Retrospective case report *(Jan 2003–Dec 2009)*	498	Overall rate 22% (n = 109) Insulin group 25% (n = 82) Insulin + non-insulin group 26% (n = 14) Sulphonylurea group 12% (n = 12) Non-sulphonylurea non-insulin group 5% (n = 1)	32–68% dependent on treatment group	65% were on insulin. 11% insulin and non-insulin agents. 19% sulphonylurea +/- other non-insulin agents. 4% non-sulphonylurea, non-insulin agents. Insulin therapy highlighted as a risk factor for recurrent hypoglycaemia among T2DM. Age and predisposing co-morbidities identified as predictors of increased long-term mortality. Three hypoglycaemic related deaths during follow-up.
Fitzpatrick & Duncan (2009)	Systematic review *(1998–2006)*	N/A	2–7% < 48 hrs	Not reported	Unable to determine if OHAs were the direct cause of certain repeat hypoglycaemic episodes. Recommends conservative management and transportation of patients on OHAs.
Walker et al. (2006)	Prospective cohort study *(Dec 2002–Mar 2003)*	38	3% (n = 1)	Not reported	12 patients (32%) reported previous SHE in previous six months. Little existing research on follow-up of patients treated by ambulance services. Patients reported satisfaction with referral pathway. Discussed possible predictors for RHE, such as advanced age.
Mattila et al. (2004)	Prospective observational study *(Feb 2001–Dec 2001)*	69	0% 24 hr re-contact 32.5% (n = 13) 3-month re-contact 55% (n = 22) reported a hypoglycaemic episode in 3-month follow-up	89.9%	76.8% SHE happened at patient’s home. 36.2% (n = 25) consumed alcohol in previous 48 hrs. 34.8% reported hypoglycaemic episode in previous 2 months. No background variables able to predict the need for EMS in the 3-month follow-up. Report up to 70% SHE happen during sleep or without warnings.
Cain et al. (2003)	Prospective observational study *(Aug 2000–Jun 2001)*	220	25.9% (n = 57) of all SHE patients 27.6% (n = 40) of not transported 22.7% (n = 17) of transported patients	66%	Authors state previous studies show higher re-contact for those not transported. Patients refused transport as opposed to referral by paramedics due to EMS system set up. Authors found no higher incidence of repeat hypoglycaemia among over 65s, despite previous studies reporting such. Highlighted need for written instructions and primary care follow-up. Repeat episodes common but not within 48 hrs. Not transporting insulin dependent patients appears safe.
Lerner et al. (2003)	Prospective case series *(Aug 1995–Jan 1998)*	36	2.63% (n = 1) 7.9% (n = 3) when including patients that self-treated recurrent hypoglycaemia	Not reported	Two patients reported recurrent hypoglycaemia within 48 hrs following discharge but self-treated and did not require EMS. One patient was found unresponsive the following morning after discharge with hypoglycaemic encephalopathy. 43% of patients were inappropriately discharged which included the two patients that self-corrected recurrent hypoglycaemia. Only 54% of patients followed discharge instructions and informed their primary care provider.
Carter et al. (2002)	Prospective cohort study *(Mar 1999–Apr 2000)*	60	4.88% (n = 2) refused transport group 5.26% (n = 1) transported group	68% (n = 41)	Patients over 65 more likely to re-contact EMS. Older patients more likely to be transported.
Anderson et al. (2002)	Retrospective case report *(1995–1998)*	1148	4.7% (n = 46) re-contact for recurrent hypoglycaemia only 2.99% < 72 hrs EMS 10.84% < 72 hrs all H/C	84% (n = 968)	Pre- and post-CBG not an accurate predictor for ED transport requirements. < 1% (n = 9) of all cases treated and left at home were admitted to hospital within 24 hrs for recurrent hypoglycaemia. Authors conclude possible to design a system to treat and leave while keeping adverse effects at an acceptably low level.
Socransky et al. (1998)	Retrospective case report *(Jan 1995–Jul 1995)*	374: patients; 571: 911 calls	6.1% (n = 25) not transported group 4.6% (n = 7) transported group	72.2% (n = 412)	Older patients tended to be more likely transported to ED. Rates of relapse did not differ significantly between those transported to ED and those not. 93.9% successful treatment and discharge (no relapse known). No deaths or ICU admission among the relapse group. Older patients and those on OHAs represent risk groups.
Mechem et al. (1998)	Prospective cohort study *(May 1996–Dec 1996)*	103	9% (n = 9) < 72 hrs	Not reported	91% (n = 94) had no recurrence of symptoms. Authors recommend transportation of insulin dependent diabetics, even if successfully treated at scene.

Note: CBG: capillary blood glycose; DM = diabetes mellitus; ED = emergency department; EMS = emergency medical services; H/C = healthcare; ICU = intensive care unit; OHA: oral hypoglycaemic agent; RHE: recurrent hypoglycaemic episode; SHE: severe hypoglycaemic episode; T1 = type 1; T2 = type 2.

## Discussion

### Non-transport/treatment at home rates

As could be expected, the non-transport rates in the studies were high, given this clinical presentation has been identified as suitable for pre-hospital management for some time (Ellis, 2002). The mean non-transport rate for the 11 studies that reported such figures was 60%, with the highest, a study from Finland, describing a figure of just under 90% and another from a UK population reporting 86% (Mattila, Kuisma, & Sund, 2004; Sampson et al., 2017). However, a small number of papers did report considerably lower figures, putting them at contrast with the general trend (Table 2).

**Table 2.** Discharge/non-transport figures.

Article	Discharge at scene/referral/non-transport rates (%)
Sinclair et al. (2018)	29.70
Sampson et al. (2017)	86.20
Bell & Fitzpatrick (2016)	38.20
Tohira et al. (2015)	29.00
Hatting & Mikkelsen (2015)	50.00
Khunti et al. (2013)	70.00
Parsaik et al. (2013)	37.60
Mattila et al. (2004)	89.90
Cain et al. (2003)	66.00
Carter et al. (2002)	68.00
Anderson et al. (2002)	84.00
Socransky et al. (1998)	72.20
**Mean**	**60.07**
**Median**	**67.00**

The lowest non-transport rate reported by the reviewed studies returned a figure of 29% (Tohira et al., 2015). This paper also stood out as it was the only study included that was carried out in an Australian population. Differing levels of autonomous practice, service protocols and training could account for some of this variance from European and North American studies.

Two other studies reported non-transport figures below 50%, although one of these was a short-cut literature review which only detailed original figures briefly (Duncan & Fitzpatrick, 2016). The third was a retrospective study on long-term outcomes for those patients requiring EMS attendance (Parsaik, Carter, Myers, Basu, & Kudva, 2013). However, this study only examined those patients with type 2 diabetes, therefore limiting the generalisability of this figure.

### Ambulance service referral

The earlier studies which reported on EMS treatment and non-transportation of patients show little if any referral pathways or safety netting (Carter, Keane, & Dreyer, 2002; Mechem, Kreshak, Barger, & Shofer, 1998). Until more recent publications, the literature reveals a practice of EMS recommending the patient arrange a follow-up appointment with their primary care provider (Anderson et al., 2002; Cain, Ackroyd-Stolarz, Alexiadis, & Murray, 2003). While authors acknowledged the importance of such follow-up and recommended structured pathways for non-transported patients, there remained inconsistent and informal procedures for many EMS providers in arranging this (Fitzpatrick & Duncan, 2009).

Only four articles that discussed specific diabetic hypoglycaemic referral pathways in detail were identified, all based on UK models. Outside of the UK, mention is made of services encouraging primary care contact, but no specific pathways were noted (Lerner, Billittier, Lance, Janicke, & Teuscher, 2003; Mattila et al., 2004). The earliest article saw a UK ambulance service referring patients following a hypoglycaemic episode to a diabetes specialist nurse (DSN) led service (Walker, James, Bannister, & Jobes, 2006). A similar pathway was set up by Leicester Primary Care Trust where patients were likewise referred to a DSN led service for review (James, Fairfield, De Groot, & Jackson, 2013). This study claimed that given the low numbers of hospital admissions by the DSN team, patients could be successfully managed in the community if referred by ambulance clinicians.

The literature discussed a range of referral or primary care notification methods by EMS providers, as well as highlighting that some make no referrals and leave follow-up to the patient’s discretion. No study clearly showed referral to a particular healthcare provider reduced adverse incidents or ambulance service re-contacts; however, qualitative data collected from patients showed those benefiting from specific referrals felt much better placed to avoid future hypoglycaemic episodes (Walker et al., 2006). Given the positive patient feedback from specialist referrals and large inconsistencies in follow-up when this is made only to a patient’s GP, a specific pathway seems the most appropriate.

### Re-contact rates

**Figure 2:**
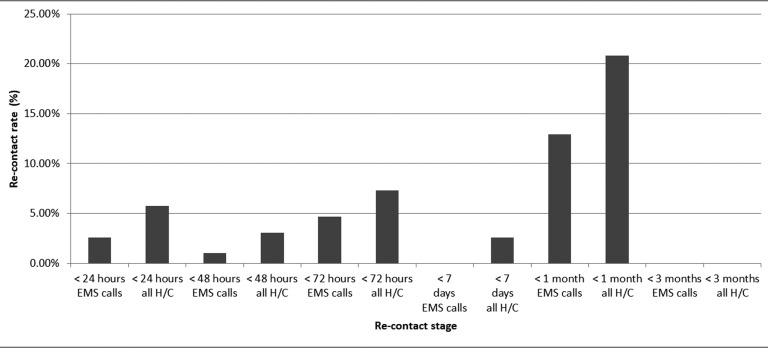
Figure 2. Re-contact rates for patients not transported.

Note: EMS = emergency medical services; H/C = healthcare. No data for < 7 days EMS, < 3 months both.

**Figure 3:**
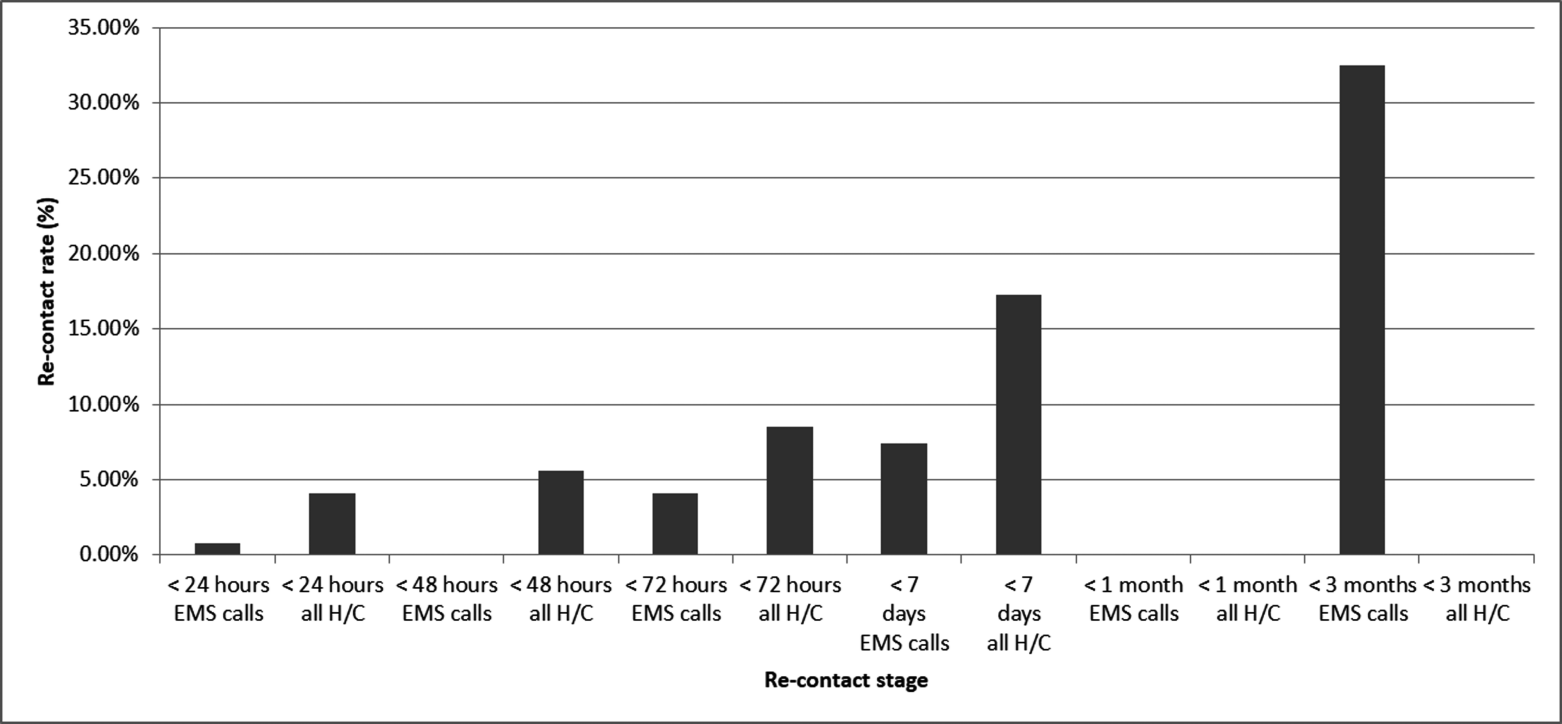
Figure 3. Re-contact rates of all hypoglycaemic patients.

Note: EMS = emergency medical services; H/C = healthcare. No data for < 48 hours EMS, < 1 month both, < 3 months all H/C.

There was virtually no consistency to the presentation of data surrounding re-contact rates, with certain papers choosing to report rates of only those that were left at scene and others the overall rates from all calls. The time scales for the reported rates varied considerably, with some reporting the re-contact data in the acute stages of 24, 48 or 72 hours following the initial event, while others described figures up to three months later. This meant that analysis of the data at a certain time point, such as 72 hours after an initial event, may have included very few studies, thus limiting the reliability of any calculations on central tendency. It did however provide a wide range of points from which to identify trends (Figures 2 and 3). There also existed variance between the papers when reporting whether a re-contact was via EMS or other healthcare provider, such as primary care or self-presentation to an emergency department.

The re-contact figures reported from the studies reflect the view of many authors that while re-contacts occur, they do not occur frequently in the acute phase and a significant proportion of these will be from unrelated, subsequent episodes (Cain et al., 2003). The community management of clinically suitable patients appears appropriate, if specialist teams and primary care can facilitate a reasonable time frame for follow-up.

Many of the articles also attempted to identify what patient groups were represented by those re-contacting EMS or other healthcare services and these are now thematically discussed.

### Type 1 versus type 2

Five of the identified papers specifically discussed findings between type 1 and type 2 diabetes, which included both prospective and retrospective studies. Between these studies there were mixed views on the influence that the diabetes type should have on the attending clinician’s pre-hospital transport decisions.

Anderson et al. (2002) retrospectively studied a physician led EMS model in Copenhagen and the data analysed showed that the type of diabetes did not appear to influence the attending physician’s decision on discharge at scene. Re-contact rates for hypoglycaemia of less than 5%, along with further analysis of their severity, led the authors to conclude that this allows for an EMS system to operate pre-hospital discharge that keeps adverse events at an acceptably low level. However, the authors fail to detail a breakdown of re-contact rates between type 1 and type 2, meaning we are left to assume that with no mention of a significant variance, none was noted.

Two further papers seem to support the idea that diabetes type need not be a significant factor in influencing clinician decisions surrounding transport to hospital (Fitzpatrick & Duncan, 2009; Walker et al., 2006). Fitzpatrick’s systematic review concludes that referral follow-up should be in as short a time as possible, regardless of diabetes type, somewhat implying both type 1 and type 2 could be managed within the community setting. In addition, Walker’s paper found that insulin treated type 2 diabetic patients and type 1 were at an equal risk of severe hypoglycaemic episodes (SHEs), although the authors do not adequately address diabetes managed by diet or oral medications.

Other research argues that diabetes type should be a consideration when not transferring patients to an emergency department (ED). One such study is Hatting’s paper, which reported a mix of type 1 and type 2 re-contacting EMS within 24 hours; however, of these patients, the subsequent hypoglycaemic episodes were minor in those with type 1 diabetes compared to the one patient with type 2. Reasons for consideration of diabetes type are discussed more clearly by Khunti et al. (2013) where it was noted that patients who had a diagnosis of type 2 diabetes and were not prescribed insulin had a higher transport rate to EDs. However, this is not solely attributed to the pathophysiology of type 2 diabetes, but rather this patient group is more likely to have a greater number of comorbidities and be older.

No study produced strong reasoning or evidence for transporting post-hypoglycaemic diabetic patients based on type alone, and it thus appears acceptable to manage both in the community. Some caution was identified surrounding those patients with type 2 diabetes; however, it appears this is more likely due to a range of factors in this group.

### Insulin treated versus non-insulin treated diabetes mellitus

One of the earliest EMS hypoglycaemia studies appeared at odds with what is now common UK paramedic practice, and recommended all insulin treated patients that experience a SHE are transported to EDs (Mechem et al., 1998). Mechem’s work was limited to include only insulin treated patients and therefore comparison with those on other treatment regimens is not possible within that study population.

Subsequent studies have since identified that the frequency of repeat hypoglycaemic episodes in insulin treated patients requiring EMS attendance is not as concerning as Mechem et al. (1998) suggested. Miller et al. (2001) found that repeat episodes of hypoglycaemia experienced by those attending a local clinic as outpatients were comparable to those of patients treated by EMS. A further study later showed that there was little difference between the re-contact rates of those insulin treated patients that were transported to an ED or those that remained at home following EMS treatment (Cain et al., 2003).

Two UK studies carried out by Khunti et al. (2013) and Sampson et al. (2017) explore the safety of discharging recovered diabetic patients by pre-hospital clinicians. Both vary from earlier literature published from North America in that these include clinician led non-transport decisions as opposed to exclusively patient refusals to travel. Khunti et al. (2013) record that diabetic patients on insulin represent nearly three quarters of hypoglycaemic calls, usually have lower blood glucose and are suffering a more severe hypoglycaemic episode. Despite this, these patients were found to be 69% less likely to be transported to an ED. Of those included in Khunti’s study, non-insulin treated patients were typically older and had a higher prevalence of co-morbidities, making them more susceptible to health complications and a poorer response to treatment. However, Sampson’s study, while supporting the conclusions that elderly patients and those with multi-morbidity were at greater risk, highlighted that adverse incidents were more likely among those on insulin therapy.

The authors, with the exception of the very earliest, agree that not transporting appropriate insulin treated patients is safe. However, there was no clear evidence as to why some authors recommended transport for non-insulin treated patients and it may simply be a risk averse approach in the absence of findings definitively showing its safety. As for diabetes type, it seems prudent to conclude that a holistic clinical picture needs to be considered, as opposed to recommending insulin treatment as the only safe sub-group suitable for non-transport.

### Sulphonylureas

Despite the reported risks of repeat hypoglycaemic episodes in patients prescribed sulphonylureas, pre-hospital EMS re-contact rates are considerably higher among those patients treated by insulin, although it should be noted that initial encounters are also much higher compared to this group (Fitzpatrick & Duncan, 2009; Sampson et al., 2017). Parsaik et al. (2013) report on the re-contact rates for each treatment group and again show re-contact rates that are lower for the sulphonylurea group compared to insulin. Parsaik’s article neglects to state the time scales applied to the figures, so it is impossible to discern whether re-contacts for one particular group are in the acute phase, or many weeks later. For this reason it is unclear if re-contacts are the result of an inappropriate pre-hospital discharge or simply a second, unrelated event a considerable amount of time later.

There is an established acceptance that the use of sulphonylureas puts patients at an increased risk of acute reoccurrence of hypoglycaemia and hospital admission is usually recommended because of this (Bailey & Krentz, 2010; Harrigan, Nathan, & Beattie, 2001). However, there remains disagreement about how long this period should be, and even how long after the initial episode the patient remains at risk. Fitzpatrick’s systematic review states that the literature currently suggests an observation period lasting no less than 24 hours, yet James’s works shows there remains a risk even outside of this window. Despite this apparent risk, Sinclair, Austin and Froats (2018) demonstrated that sulphonylurea use was not an independent predictor of repeat events in those patients attended by EMS.

A number of studies demonstrated the majority of patients prescribed oral hypoglycaemic agents, and specifically sulphonylureas, are an older population with a higher number of comorbidities (Dunning, 2014; Khunti et al., 2013; Parsaik et al., 2013; Sampson et al., 2017). These demographic findings may highlight that sulphonylureas alone are not an automatic risk factor for this group, but rather the patient’s pre-existing conditions, although none of the studies examine this possibility specifically. It could be the case, as for non-insulin treated patients, that transport recommendations are made to reduce the risk of adverse incidents in the absence of clear evidence demonstrating safety.

### Patient age

One of the earliest studies recommended a greater emphasis should be placed on transporting older patients to EDs, regardless of pre-hospital treatment outcomes (Socransky, Pirallo, & Rubin, 1998). This is further documented in four prospective studies which highlight advanced age as a risk factor for hypoglycaemic episodes (Cain et al., 2003; Carter et al., 2002; Sampson et al., 2017; Walker et al., 2006). Of these studies, three discuss repeat EMS contacts after the initial attendance and, surprisingly, they do not report a higher instance of repeated contacts among the older population (Cain et al., 2003; Carter et al., 2002; Sampson et al., 2017). This is likely because all the studies demonstrated a higher proportion of older patients being transported to hospitals, one study reporting 61% for patients over 65 years old versus 24% below. The similar re-contact results between the age groups could demonstrate that ambulance clinicians are accurately identifying patients that require ED treatment, particularly given that of those older patients transported, a high proportion are subsequently admitted (Socransky et al., 1998).

### Comorbidities

Parsaik’s retrospective study of type 2 diabetic patients showed an increased risk of mortality in those patients with a number of comorbidities that required EMS assistance for a SHE. The study also reported that major causes of death were respiratory and cardiovascular illnesses, a finding which had been identified previously (Evans, Ogston, Emslie-Smith, & Morris, 2006). These respiratory and cardiovascular causes of death identified by Parsaik could be linked to findings in Khunti’s study which demonstrated a higher respiratory rate as a positive predictor in those requiring transport to hospital. Further studies acknowledged comorbidities as a risk factor in those patients experiencing a hypoglycaemic episode pre-hospital but acknowledge higher hospital transport rates and admissions in this group may also be due to advanced age or treatment types (Cain et al., 2003; Sampson et al., 2017; Sinclair et al., 2018).

As for elderly patients, those with increased comorbidities showed increased transportation and admission rates; however, specific re-contact figures were not detailed. It is likely a large proportion of those patients reporting multi-morbidity will also be older and thus it is difficult to say if age, frailty or comorbidities are the most influencing risk factors in clinician decision making.

### Limitations

The study was limited by the researcher’s own access to published studies, as noted; only those freely available and accessible through existing database privileges were included. However, these were relatively small in number, with the overwhelming majority identified during screening retrieved for full appraisal. While only UK treat, leave and refer protocols were screened, these were ultimately not included due to absence of re-contact data. However, availability of international ambulance services procedures may have helped better identify reasons for referral/re-contact rate variances across the studies and this is an area which would require further exploration.

## Conclusion

The literature demonstrates that ambulance clinicians appropriately treat hypoglycaemia in the community and confidently identify those requiring further assessment at EDs. However, due to the very nature of diabetes and individual compliance with treatments, repeat episodes will and do occur, regardless of community or ED management, but these are rarely in the acute phase.

Checklists/protocols were shown by Tohira et al. (2015) to be inferior to holistic assessment by clinicians in identifying those suitable to be managed in the community setting and the other literature appears to support that. While some, such as older patients and those with numerous comorbidities, were identified as higher risk, there are still many within these groups that can be appropriately managed in the community. With increased clinician education, alongside the development of specialist roles such as community and advanced paramedics, it could be expected that more patients contacting the ambulance service following a hypoglycaemic episode will be able to benefit from community care while keeping re-contacts at an acceptably low level.

## Conflict of interest

None declared.

## Funding

None.
